# Development of Chicken Embryos in Double-Yolk Eggs: Fertility, Hatchability, Embryo Malposition and Time of Embryonic Mortality

**DOI:** 10.3390/ani13182931

**Published:** 2023-09-15

**Authors:** Dorota Banaszewska, Angelika Kasianiuk, Barbara Biesiada-Drzazga, Urszula Zaremba

**Affiliations:** Faculty of Agrobioengineering and Animal Husbandry, Siedlce University of Natural Sciences and Humanities, Prusa 14, 08-110 Siedlce, Poland; dorota.banaszewska@uph.edu.pl (D.B.); angelika240698@wp.pl (A.K.); barbara.biesiada-drzazga@uph.edu.pl (B.B.-D.)

**Keywords:** Pasgar©score, health assessment, laying hens, double-yolk

## Abstract

**Simple Summary:**

Due to lower parameters such as fertility rate and hatchability rate in comparison with single-yolk eggs, double-yolk eggs are not used for incubation in commercial poultry production. For this reason, double-yolk eggs are rarely the subject of research, and knowledge of embryonic development in this type of egg remains scarce. In this study, three types of chicken eggs (single-yolk eggs, double-yolk eggs with one developing embryo, and double-yolk eggs with two developing embryos) were compared in terms of embryonic mortality during incubation and chick quality. Embryonic mortality differed between single-yolk and double-yolk eggs at all stages of incubation. In the case of double-yolk eggs, embryonic mortality did not differ only in the initial stage of incubation. Despite the fact that two embryos in a single egg during incubation developed to a fairly advanced stage, no chicks were obtained from this type of egg. No differences were shown in chick quality between chicks from single-yolk eggs and double-yolk eggs with one developing embryo, but the weight of chicks from double-yolk eggs was significantly higher. Embryo malposition was more common in double-yolk eggs with two developing embryos. The study contributes to knowledge of embryo development and mortality in double-yolk eggs.

**Abstract:**

Fertility rate and hatchability rate are low for all types of double-yolk (DY) eggs in comparison to single-yolk eggs (SY), but these parameters also depend on the number of developing embryos in the egg. The hatchability rate of double-yolk eggs containing two developing embryos (DY2F) is vastly lower than in the case of double-yolk eggs containing only one embryo (DY1F). The aim of the study was to determine the differences between egg fertility rate, hatchability rate, time of embryonic mortality, and embryo malposition during incubation in three types of eggs from Hy-Line Brown hens: SY, DY1F and DY2F. In addition, the quality of the hatched chicks was assessed using the Pasgar©score. Following a 21-day incubation, chicks were obtained from DY1F and SY eggs. No chicks were obtained from DY2F eggs, although the embryos in these eggs developed up to the late stage of incubation. Early (≤7 d of incubation), middle (8–14 d), and late (≥15 d) embryonic mortality was significantly higher in DY eggs than in SY eggs. The embryonic mortality rate during early incubation was the same for DY1F and DY2F eggs, but middle and late embryonic mortality were significantly higher for DY2F eggs. Based on evaluation of embryo position according to Landauer, only three types of malposition that could potentially lead to embryonic death were noted. There were fewer malpositioned embryos in double-yolk eggs containing one embryo. Quality assessment of chicks (Pasgar©score) showed no differences between chicks hatched from eggs containing one yolk and those hatched from double-yolk eggs with one developing embryo, but chicks from double-yolk eggs were significantly heavier. The results of the research will contribute to a better understanding of the development and mortality of embryos in double-yolk eggs.

## 1. Introduction

Disturbances during the typical time of ovulation in birds can lead to simultaneous ovulation of two egg follicles, resulting in double-yolk eggs (DY) [1,2]. DY eggs are relatively rare in nature but are encountered across diverse avian taxa [3,4,5]. Double-yolk eggs are relatively common in commercial poultry production [6]. DY eggs are more common in layers that have just begun laying than in mature hens [6,7]. According to current knowledge, the occurrence of DY eggs may be influenced by a number of factors: age, diet, photostimulation, and selection for multiple ovulations, which suggests a genetic effect [6,7,8,9,10].

Compared to single-yolk (SY) eggs, DY eggs are characterized by higher embryonic mortality at all stages of development and by lower fertility and hatchability rates [11,12,13,14]. For this reason, this type of egg is not used for incubation in commercial poultry production. The low fertility rate in DY eggs compared to SY eggs may be linked to yolk size and maturity at the onset of laying [15] or may be due to the inclusion of premature or over-mature follicles, whose capacity to be fertilized may be reduced in comparison with a mature follicle included in the same DY egg [14]. Due to their low commercial importance, DY eggs are relatively rarely the subject of research, and therefore knowledge of embryonic development in this type of egg remains scarce [11,12,13,14]. DY eggs more often contain one fertilized yolk (DY1F) than two fertilized yolks (DY2F). DY2F eggs have much lower hatchability rates than DY1F eggs. Nevertheless, two embryos developing in a single egg can develop to relatively late stages of development. For example, embryos in DY2F eggs of pigeons, *Columba livia*, died just before hatching [16]. Rare cases of development of twin embryos have also been observed in the ostrich, *Struthio camelus* [17], turkey, *Meleagris gallopavo domesticus* [18], and emu, *Dromaius novaehollandiae* [19], but all embryos in the eggs died at the late stage of development. In chickens, attempts to obtain two chicks from one egg (DY2F) have rarely been successful, and only in the case of assisted hatching [11,12]. The causes of embryonic mortality in DY eggs are not fully known. It has been speculated that when two embryos develop in one egg, the size of the egg may limit the embryos’ ability to assume the position necessary for hatching [12]. Researchers also point out that the shell surface area may be insufficient to ensure effective respiration in the developing embryos [14,16].

The aim of the study was to provide and analyze information on the differences between fertility rate, hatchability rate, and time of embryonic mortality in DY1F, DY2F and SY eggs from Hy-Line Brown hens. In addition, the health quality of the chicks hatched from DY and SY eggs was assessed using the Pasgar©score [20]. We suspected that the quality of chicks hatched from DY eggs would be lower than that of chicks hatched from SY eggs. The position of dead embryos in DY1F and DY2F eggs was evaluated according to Landauer [21]. The analysis was aimed at establishing the time of embryonic mortality during three stages of incubation in DY and SY eggs and to determine whether embryo malposition could be associated with the higher embryonic mortality in DY eggs during incubation.

## 2. Materials and Methods

### 2.1. Egg Collection and Animal Management

The research material consisted of DY (DY1F and DY2F) and SY eggs obtained from 35-week-old Hy-Line Brown hens (*n* = 996). DY eggs were identified by illuminating a bright light through each egg (candling). By sending light through the DY egg, two yolks become visible. Hatching eggs were obtained by natural mating of Hy-Line Brown hens with roosters of the same line. There was one rooster for every 10 hens. Stocking density did not exceed 9 laying hens per m^2^ usable area. The birds were kept on deep litter in enclosed housing in accordance with the requirements given in the ‘Hy-Line Brown Management Guide’. Diets were formulated based on the management guidelines for Hy-Line laying hens (310–325 kcal/bird/day). Complete feed contained 15.55% crude protein, 0.80% lysine, 0.38% methionine, 3.91% calcium and 0.38% phosphorus in 1 kg of mix.

### 2.2. Egg Preparation and Incubation

The DY eggs obtained were individually numbered and evaluated before incubation using non-destructive methods to assess egg quality indicators: egg weight, shell quality, air cell location, number of yolks, and egg length and width. The eggs were weighed with an electronic Ohaus scale to within 0.01 g. They were weighed three times: on the day they were placed in the incubator, during the first candling (7 d of incubation), and during the second candling (14 days of incubation). The depth of the air cell was determined at 7 and 14 days of incubation using an Ovolux candling lamp and a millimeter scale. The first and second candlings were performed at 7 and 14 days of incubation, respectively. After the first candling at 7 days of incubation, only one developing embryo could be seen in DY1F, and two developing embryos could be seen in DY2F. After each candling, eggs which were clearly seen to lack a developing embryo, or in which embryonic development had stopped, were eliminated from further incubation. The fertile eggs were stored no longer than 7 days prior to incubation at 15 °C in cardboard trays with the pointed end up. They were incubated in an automatic incubator. The temperature during incubation was maintained at 37.8 °C and relative humidity at 50–60%. Incubated eggs were turned automatically, 45° every hour [22]. At 19 days of incubation the eggs were transferred to hatching baskets. The temperature in the hatcher was 37.0 °C. Relative humidity was maintained at 50–54% until hatching. At 20 days of incubation, it was increased to 70–75%, and after hatching the relative humidity in the hatcher was gradually decreased from 75% to about 53%.

### 2.3. Time of Embryonic Mortality and Malposition Assessment

Eggs from which chicks did not hatch were opened in order to assess the time of embryonic death. During evaluation of the development of dead embryos, an attempt was made to find the anatomic element that should have been present on a given day of embryogenesis [23].

Embryonic malposition was evaluated using the Landauer scale [21], which classifies chicken embryonic malposition into 7 types (designated 1–7). In describing the individual defects, the previously estimated age of the embryo was taken into account, because a given malposition may have been a normal position at an earlier stage of development [24].

### 2.4. Chick Quality Assessment

Between 1 and 2 h after hatching, dry chicks underwent qualitative analysis using the Pasgar©score [20,25] as well as quantitative measurements of body weight. The Pasgar©score used to evaluate the quality of each chick is based on five criteria: (1) navel condition (black button or leaky navel); (2) yolk sac (large residual yolk sac); (3) red hocks (red or swollen hocks); (4) abnormal beak (red beak or nostrils contaminated with albumen); and (5) low alertness. For each of the five criteria, one point was subtracted from 10, so that a score of 10 indicated chicks free of any abnormality, and 5 was the lowest possible score.

### 2.5. Statistical Analyses

All data were analyzed and visualized using R and R Studio [26]. Differences between SY and DY eggs were tested by the Student *t*-test. A generalized linear model (GLM) with binomial error distribution was used to determine differences in mortality rates of embryos between types of eggs and incubation stages. The GLM model was fitted with events (death) and non-events (died, risk–died) as a response variable, and categorical variables group (control, DY1F, DY2F) and incubation stage (early, middle, late) as covariates in the following saturated model: glm(cbind (died, risk–died) ~ group*stage, family = binomial)). Estimates of the probabilities of embryo death for each stage and group were provided by the emmeans function (emmeans package); [27]. Pairwise post hoc comparisons between groups and incubation stages were performed with estimated marginal means using the contrast function (emmeans package).

Because double-yolk eggs were analyzed, some traits were calculated not per egg but per yolk, i.e., fertility ((number of yolks with fertile germs/number of yolks in set eggs set) × 100), hatchability ((number of hatched chicks/number of yolks in set eggs) × 100) and hatch of fertile ((number of hatched chicks/number of yolks with fertile germs) × 100). Percentage embryo mortality was calculated as the number of embryos that died during one of the three mortality periods divided by the number of yolks with fertile germs × 100.

Egg length and width were measured using an electronic calliper (Stainless Hardened) to the nearest 0.01 mm and used to calculate the egg shape index (SI = width/length × 100), according to Panda [28].

## 3. Results

### 3.1. Egg Quality Characteristics

A total of 996 eggs obtained from Hy-Line Brown hens were used in the experiment, including 498 SY eggs, constituting the control, and 498 DY eggs. Means ± SD of external and internal traits of SY and DY hatching eggs are presented in Table 1. After the first candling at 7 days of incubation, unfertilized eggs were removed from the incubator, so that 460 SY eggs, 138 DY2F eggs, and 216 and DY1F eggs remained.

### 3.2. Fertility and Hatchability Traits

Fertility and hatchability traits in incubated SY, DY1F and DY2F chicken eggs are summarized in Table 2. The fertility rate of SY and DY eggs was 92.37% (460/498) and 49.4% (492/996), respectively. Hatchability of SY eggs was 86.55% (431/498), while that of DY eggs was 10.84% (108/996). There were 431 chicks hatched from SY eggs, for a “hatch of fertile” result of 93.69% (431/460), while 108 chicks were obtained from DY1F eggs, for a result of 50% (108/216). No chicks were obtained from DY2F eggs due to 100% embryo mortality.

### 3.3. The Embryo Mortality Rate and Probability of Death

**Figure 1:**
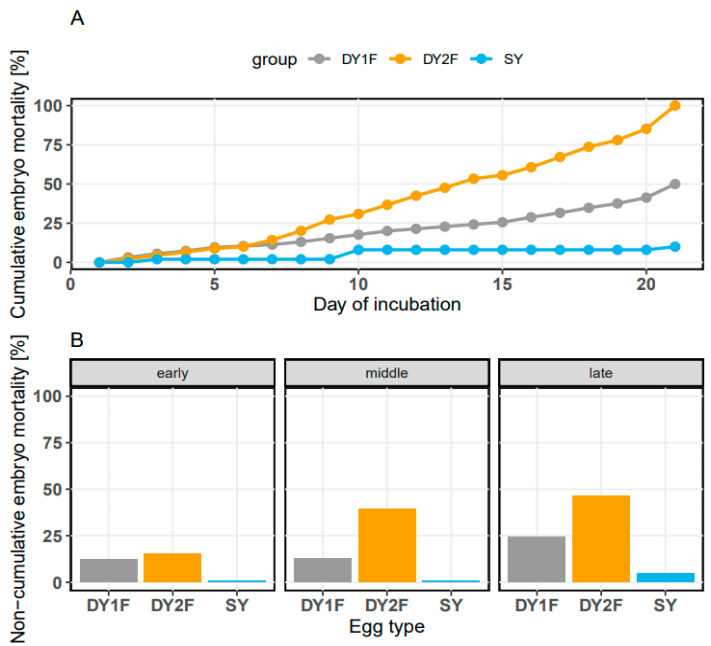
(**A**)—cumulative embryo mortality (%) of DY1F, DY2F and SY eggs (DY2F—double-yolk egg with two fertilized yolks; DY1F—double-yolk egg with one fertilized yolk; SY—single-yolk egg with fertilized yolk) between measurement days over the incubation period. (**B**)—non-cumulative embryo mortality in three incubation stages: early (1–7 days), middle (8–14 days), late (15–21 days).

**Figure 2:**
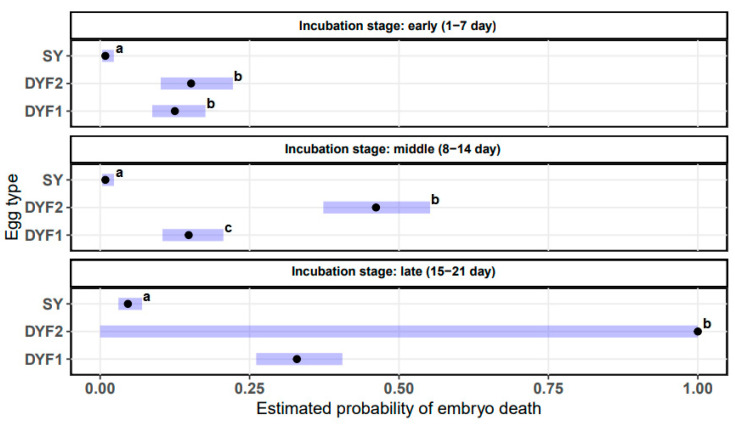
Estimated probability of embryo death in SY, DY2F, DY1F eggs (DY2F—double-yolk egg with two fertilized yolks; DY1F—double-yolk egg with one fertilized yolk; SY—single-yolk egg with fertilized yolk). Error bars are the 95% confidence limits. Confidence limits for DY2F in late stage of incubation cannot be calculated due to perfect separation. Statistical significance between the groups is shown by letters: a, b, c. In groups with the same letter, the difference between the means is not statistically significant.

The embryo mortality rate and probability of death differed between egg types and incubation stages (Table 3, Figure 1 and Figure 2). The total mortality of incubated embryos was lowest for SY eggs, at 6.31% (29/460), while in the case of DY1F and DY2F eggs it was 50% (108/216) and 100% (276/276), respectively (Figure 1A). During the initial incubation period (1–7 d), the percentage of dead embryos in SY, DY1F and DY2F eggs was 0.87% (4/460), 12.5% (27/216), and 15.6% (43/276), respectively (Figure 1B).

During this period, the probability of embryo death in SY eggs was 0.01 and was significantly (*p* < 0.001) lower than in DY1F and DY2F eggs, which was 0.13 and 0.15, respectively (Table 3). The probability of embryo death in DY1F and DY2F eggs did not differ significantly (*p* > 0.747). During the middle period of incubation (8–14 d), the percentage of dead embryos was 0.87% (4/460) for SY eggs, 13% (28/216) for DY1F eggs, and 38.8% (107/276) for DY2F eggs (Figure 1B). The probability of embryo death in SY eggs was equally low (0.01), but in DY1F and DY2F eggs it was 0.15 and 0.46, respectively. The probability of embryo death was significantly different between all types of incubated eggs (*p* < 0.001). During the final incubation period (15–21 d), the percentage of dead embryos was 4.56% (21/460) for SY eggs, 24.5% (53/216) for DY1F eggs, and 45.6% (126/276) for DY2F eggs (Figure 1B). The probability of embryo death in SY eggs was 0.05, while in the case of DY1F and DY2F it was 0.33 and 1.00, respectively. Because all embryos in DY2F eggs died during the late stage of incubation, the GLM model showed perfect separation (fitted probabilities reached 0%). This meant that confidence limits could not be calculated, and significance levels between eggs could not be compared (Table 3).

### 3.4. Embryo Malposition

Malposition of dead embryos according to Landauer [21] was observed (Table 4). Only 5.43% (15/276) of dead embryos from DY2F eggs exhibited malposition. The most common malposition in these eggs was type 1 (9/276). Types 4 (3/276) and 5 (3/276) were observed as well. In the case of DY1F eggs, malposition was observed in less than 1.38% (3/216) of dead embryos, and the only malposition type was type 5 (3/216).

### 3.5. Quality Assessment of Chicks

There were 108 chicks hatched from the 216 incubated DY1F eggs, of which six died on the second day after hatching. Among the 108 chicks, 45 were female and 63 male. There were 431 chicks hatched from the 460 incubated SY eggs (205 female and 226 male). Hatched chicks had high quality scores (Pasgar©score); the average was above 9.8 for both the SY and DY1F groups (Table 5).

## 4. Discussion

In the present study, as expected, the fertility rate of double-yolk (DY) eggs obtained from Hy-Line Brown hens, containing at least one fertilized yolk, was significantly lower than in the case of SY eggs. At the same time, the fertility rate of the DY eggs was similar to literature results. For example, a somewhat higher percentage of fertilized DY eggs was reported by Fasenko et al. [14], who incubated Hubbard Hi-Y chicken eggs from a commercial flock and obtained a 62.5% fertility rate. In that study, however, the hens were younger (30 weeks old). A somewhat lower percentage of fertilized DY chicken eggs (42.9%) was reported by Fechheimer and Jaffe [29], but a variety of breeds were used in that study, the age of the hens is unknown, and the work itself should currently be regarded as historical. A relatively low fertility rate (51.9%) was obtained by Salamon and Kent, [15] for incubated DY eggs of Aylesbury ducks, which may confirm that the rule that duck eggs have a lower fertility rate than chicken eggs applies to double-yolk eggs as well.

A low hatchability rate of incubated DY chicken eggs was recorded in the present study (DY1F). No chicks were hatched from any of the DY2F eggs, despite the fact that both embryos developed to an advanced stage during incubation, as evidenced by the fact that embryonic mortality in this type of egg was highest during the third, final stage of incubation. These results confirm the extremely low hatchability rate of DY2F eggs. For example, a low hatchability rate of DY eggs was reported by Fasenko et al. [14] for incubated DY Hubbard Hi-Y eggs. As in the present study, Fasenko et al. [14] also obtained only single chicks from DY eggs. Salamon and Kent [15] also observed a low hatchability rate in the case of incubation of fertilized DY duck eggs. Single ducklings hatched from only 2.52% of DY1F eggs, and two ducklings were obtained from 0.44% of DY2F eggs. Burke et al. [13] also reported a relatively low percentage of hatched DY eggs in two separate experiments (2.18% hatched DY1F eggs; 4.24% hatched DY1F eggs). In that study, however, the authors studied the development of single embryos in eggs containing two yolks (DY1F), so there is no information on the hatchability of DY2F eggs. Although in the present study it was not possible to obtain two chicks from DY2F chicken eggs, this was accomplished by Jeffrey [11], who incubated 152 DY eggs and obtained two chicks from one DY2F egg; however, these chicks required assistance to hatch. Monkman [12] also obtained 14 ‘twin’ chicks from 7 DY2F eggs and 37 single chicks from both DY1F and DY2F eggs, which is a relatively high (20%) hatching rate of incubated DY eggs. It should be noted, however, that like Jeffrey et al. [11], Monkman [12] assisted the chicks in hatching by attempting to prevent the embryos from suffocating in the eggs, which most likely reduced embryonic mortality during the final stage of incubation and increased the chance of obtaining two chicks from one egg. It should be stressed that in the present study the chicks were not assisted during hatching, which may have contributed to the fact that no chicks hatched from DY2F eggs. Two embryos in DY2F eggs can develop to a relatively advanced stage of incubation. The increasing size of embryos may limit their movement in the egg, impairing the development and positioning of twin embryos in DY2F eggs, and is most likely the reason for the drastically reduced hatchability rate of DY2F eggs.

In SY eggs of avian species, there are two critical periods during incubation when mortality rates peak: one in early embryonic life and the other shortly before hatching [24,25]. High embryonic mortality in DY eggs is associated with significantly higher mortality during the early, middle and late incubation periods in DY eggs in comparison to SY eggs, which is confirmed by other authors [14,15]. The present study also showed high mortality rates for embryos from DY eggs during the three stages of incubation. In the initial stage of incubation (1–7 days), DY1F and DY2F eggs did not differ significantly in terms of embryonic mortality. Differences in embryonic mortality between DY1F and DY2F eggs were significant beginning in the second stage of incubation (8–14 days), when a sharp increase in embryonic mortality from DY2F eggs was observed. This may suggest that the rapid growth of two embryos in a single egg and the limited surface area of the egg containing two embryos may contribute to increased mortality in double-yolk eggs by impeding respiration and limiting the correct positioning of the embryos in the final stage of incubation. Salamon and Kent [15] reported that early mortality in duck eggs was higher in DY1F eggs (31.58%), while late mortality was higher in DY2F eggs (70.38%). In the present study, the two embryos in DY2F eggs developed normally until the late stage of incubation (15–21 days), when embryonic death took place. Malposition of embryos in eggs according to Landauer [21] was noted in only a few of the dead embryos. This suggests that the high mortality in DY eggs may be due to an insufficient shell surface area, which would prevent effective respiration in the period preceding hatching [12,14,16]. On the other hand, chicks hatched from DY1F and SY eggs did not differ significantly in quality according to the Pasgar©score. The average weight of chicks from DY eggs was higher than for chicks hatched from SY eggs, which is consistent with results reported by other authors [13,14]. The higher weight of chicks from DY eggs may be due to the fact that these chicks internalize the other yolk [14].

## 5. Conclusions

No chicks were obtained from DY2F eggs, although the embryos in these eggs developed up to the late stage of incubation. The probability of embryonic mortality was significantly higher in DY eggs than in SY eggs during early, middle and late incubation stages. There were fewer malpositioned embryos in double-yolk eggs containing one embryo. Quality assessment of chicks (Pasgar©score) showed no differences between chicks hatched from SY and those hatched from DY eggs, but chicks from double-yolk eggs were significantly heavier. The results of the research will contribute to the knowledge of embryo development and mortality in Hy-Line Brown DY eggs.

## Figures and Tables

**Table 1:** Mean (+SD) for egg quality characteristics and hatchling mass in SY and DY (pooled DY2F DY1F) Hy-Line Brown chicken eggs.

Egg trait	DY	SY	*p*-Value
*n* (eggs)	498	498	
Egg weight, g	75.14 ± 6.04	62.4 ± 2.51	<0.0001
Egg weight (1st candling)	72.44 ± 6.04	59.5 ± 2.48	<0.0001
Egg weight (2nd candling)	67.39 ± 6.01	56.1 ± 2.46	<0.0001
Egg length, mm	61.3 ± 3.22	55.3 ± 3.01	<0.0001
Egg width, mm	46.68 ± 1.25	44.9 ± 2.03	<0.0001
Egg shape index, %	1.31 ± 0.06	1.23 ± 0.11	<0.0001
Air cell height, mm	2.53 ± 0.54	2.92 ± 0,34	<0.0001
*n* (hatchlings)	108	431	
Hatchling weight 48 h after hatch, g	48.63 ± 4.90	40.03 ± 3.80	<0.0001

SY—single-yolk egg with a fertilized yolk; DY1F—double-yolk egg with one fertilized yolk; DY2F—double-yolk egg with two fertilized yolks.

**Table 2:** Fertility and hatchability traits in incubated DY (DY1F, DY2F) and SY Hy-Line Brown chicken eggs.

Egg Type	DY2F	DY1F	SY	DY	*p*-Value
Fertility rate %			92.74	49.4	<0.0001
Hatchability %			86.89	10.84	<0.0001
Hatch of fertile %	0	50.00	93.69	21.95	<0.0001

SY—single-yolk egg with a fertilized yolk; DY1F—double-yolk egg with one fertilized yolk; DY2F—double-yolk egg with two fertilized yolks.

**Table 3:** Estimated probabilities of embryo death +SE (standard error) for each group (SY, DY1F, DY2F) and incubation stage (early, middle, late).

Egg Type	*n* Embryo (Alive)	Probability of Embryo Death	SE	LCL	UCL	Contrast	*p*-Value
	Incubation stage = early (1–7 day)						
SY	456	0.01	0.00	0.00	0.02	SY/DY1F	<0.0001
DY1F	189	0.13	0.02	0.09	0.18	DY1F/DY2F	0.7473
DY2F	233	0.15	0.03	0.10	0.22	DY2F/SY	<0.0001
	Incubation stage = middle (8–14 day)						
SY	452	0.01	0.00	0.00	0.02	SY/DY1F	<0.0001
DY1F	161	0.15	0.03	0.10	0.21	DY1F/DY2F	<0.0001
DY2F	126	0.46	0.05	0.37	0.55	DY2F/SY	<0.0001
	Incubation stage = late (14–21 day)						
SY	431	0.05	0.01	0.03	0.07	SY/DY1F	<0.0001
DY1F	108	0.33	0.26	0.26	0.41	DY1F/DY2F	
DY2F	0	1.00	0.00	0.00	100	DY2F/SY	

SY—single-yolk egg with a fertilized yolk; DY1F—double-yolk egg with one fertilized yolk; DY2F—double-yolk egg with two fertilized yolks. UCL and LCL represent upper and lower confidence limits.

**Table 4:** Fertility types of embryonic malposition in dead embryos in DY2F and DY1F.

Egg Type	DY2F	DY1F
Total Number of Malpositioned Embryos in Incubated DY Eggs (from Day 15 of Incubation)	*n* = 15	*n* = 3
1—Head between thighs	9	-
2—Head in small end of egg (opposite air cell)	-	-
3—Head under left wing	-	-
4—Head in normal position but rotated with beak pointed away from air cell	3	-
5—Feet over head	3	3
6—Beak (or head) over right wing	-	-
7—Embryo perpendicular to main axis of egg	-	-

DY1F—double-yolk egg with one fertilized yolk; DY2F—double-yolk egg with two fertilized yolks.

**Table 5:** Chick quality assessment using the Pasgar©score which hatched from DY1F and SY eggs.

Egg Type Number of Hatched Chicks	DY1F *n* = 108	SY *n* = 431
Navel condition: (black navel; leaky navel) %	3.70 (4)	3.01 (13)
Yolk sac (large residual yolk sac) %	0.93 (1)	0.23 (1)
Red hocks (red or swollen hocks) %	0.93 (1)	0.23 (1)
Abnormal beak (red beak or nostrils contaminated with albumen) %	0.93 (1)	0.23 (1)
Low alertness %	9.26 (10)	8.12 (35)
Mean + SE Pasgar©score:	9.86 + 0.03 SE	9.88 + 0.02 SE

SY—single-yolk egg with a fertilized yolk; DY1F—double-yolk egg with one fertilized yolk.

## Data Availability

The data presented in this study are available on request from the corresponding author.
